# A pilot study examining hemomania behaviors in psychiatry outpatients engaged with nonsuicidal self‐injury

**DOI:** 10.1002/brb3.3475

**Published:** 2024-04-09

**Authors:** Ali Kandeger, Omer Faruk Uygur, Emine Yavuz Ataslar, Furkan Cınar, Yavuz Selvi

**Affiliations:** ^1^ Department of Psychiatry Selçuk University Konya Türkiye; ^2^ Department of Psychiatry Atatürk University Erzurum Türkiye; ^3^ Department of Psychiatry Etlik City Hospital Ankara Türkiye

**Keywords:** hemomania, impulsivity, nonsuicidal self‐injury, suicidality

## Abstract

**Background:**

This study aims to conduct the first‐ever evaluation of our previously proposed behaviors of “hemomania” in individuals engaged with nonsuicidal self‐injury (NSSI).

**Methods:**

The study encompassed 130 outpatients engaged with NSSI who applied at the psychiatry outpatient clinic. NSSI behaviors were assessed using the Inventory of Statements About Self‐Injury, while psychiatric diagnoses were evaluated using the Structured Clinical Interview for DSM‐5 Disorders‐Clinician Version. Subsequently, participants completed the Depression Anxiety Stress Scale‐21 and Short Form of Barratt Impulsiveness Scale.

**Results:**

The prevalence of at least one hemomania behavior including seeing blood, tasting blood, bloodletting, and blood‐drinking was observed to be 43.1% in individuals with NSSI. When participants were divided into two groups, individuals with hemomania exhibited: (1) a higher incidence of psychiatric comorbidities, increased suicide attempts, and more severe symptoms of depression, anxiety, stress, and impulsivity, (2) higher comorbidity rates of borderline personality disorder, body‐focused repetitive behaviors, and dissociative disorders, and (3) elevated frequencies of certain NSSI behaviors, including cutting, biting, needle‐ticking, and carving, compared to those without.

**Conclusion:**

Hemomania could be considered a specific impulse control disorder, characterized by heightened impulsivity and a persistent urge to obtain one's own blood. However, further studies are needed to validate this hypothesis.

## Graphical abstract

This pioneering study investigates hemomania behaviors involving the urge to obtain one's own blood in psychiatry outpatients engaging with NSSI. Among 130 outpatients, 43.1% exhibited at least one hemomania behavior, as shown in the prevalence diagram. Those with hemomania displayed heightened psychiatric comorbidities, increased suicide attempts, and severe symptoms of depression, anxiety, stress, and impulsivity. Elevated rates of certain NSSI behaviors were observed. These findings suggest hemomania's potential classification as an impulse control disorder, warranting further validation and exploration.

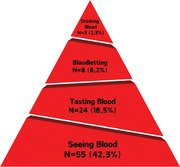

## 1 INTRODUCTION

Nonsuicidal self‐injury (NSSI) is categorized under “Other Conditions That May Be a Focus of Clinical Attention” in the DSM‐5 (American Psychiatric Association, 2013). It refers to intentional self‐inflicted harm to the body that may cause bleeding, bruising, or pain (e.g., cutting, burning, hitting) without suicidal intent (Klonsky & Muehlenkamp, 2007; Nock & Favazza, 2009). If NSSI is repeated and impairs an individual's functioning, it has been emphasized that investigation of the diagnosis of nonsuicidal self‐injury disorder (NSSID) should be investigated. This diagnosis has been included under the “Conditions for Further Study” section in the DSM‐5 (Zetterqvist, 2015).

NSSI has emerged as a significant public health concern, particularly given its alarmingly increasing prevalence among adolescents and young adults (Muehlenkamp et al., 2012). Accumulated data consistently indicates a higher prevalence of NSSI among women (Ose et al., 2021; Swannell et al., 2014). A recent systematic review found a prevalence of 38.9% in young adults, 4%–23% in adults, 11%, and 21.5% in psychiatric outpatients and inpatients, respectively (Arnold et al., 2022; Cipriano et al., 2017; Plener et al., 2016). Self‐cutting is the most common form of NSSI, although individuals who engage in NSSI often utilize multiple methods (Favazza & Conterio, 1988; Swannell et al., 2014). Furthermore, NSSI frequently co‐occurs with borderline personality disorder, dissociative disorders, mood disorders, eating disorders, substance use disorder, and symptoms of anxiety, depression, and stress (Christoforou et al., 2021; Ose et al., 2021).

The desire to see blood is a behavior that has received insufficient attention in scientific research and is relatively less known. Prior to empirical investigations, references to the desire to see blood were made in popular media, song lyrics, and quotes from books. In 1994 and 1996, case series were presented featuring women who engaged in self‐cutting, some of whom reported that they cut themselves with the intention of seeing blood, and that observing blood was associated with relief and a decrease in tension (Himber, 1994; Solomon & Farrand, 1996). The first empirical study on this topic reported that 47% of women with self‐mutilation reported that seeing blood was comforting (Favazza & Conterio, 1989). Among these participants, a considerable number (84.8%) revealed that observing their own blood aided in reducing their tension, while 72.7% reported that it helped to calm down (Glenn & Klonsky, 2010).

Behaviors associated with a desire for blood extend beyond the mere desire to see it. Upon examination of the literature, it becomes apparent that there exist other more detrimental behaviors that exhibit overlapping symptoms with a desire to view blood. The precise position of these behaviors within the psychiatric literature remains indeterminate (Kandeğer et al., 2021). One of them, bloodletting, is a form of self‐harm that has been documented in the psychiatric literature, particularly in cases of borderline personality disorder and bulimia nervosa (Parkin & Eagles, 1993; Patel et al., 2006; Warren et al., 1998). This practice involves individuals draining their own blood using a syringe or cannula and can lead to factitious anemia or even death. The individuals who engage in bloodletting commonly report that their main objectives are to increase their overall sense of well‐being, reduce distress, and obtain relief from their symptoms (Dursun et al., 2010; Kandeğer et al., 2021).

Although blood‐drinking behavior is primarily associated with myths and vampire folklore, a limited number of clinical cases involving blood‐drinking have been identified (Mac Suibhne & Kelly, 2010). In a study, Favazza and Conterio (1989) reported that 25% of self‐mutilating individuals had tasted their own blood. While blood‐drinking has been observed in association with some psychiatric disorders, there has yet to be a systematic investigation of this phenomenon (Sakarya et al., 2012). However, in a recent report, we described two cases of impulse control disorder characterized by blood‐drinking behavior and proposed the term hemomania to define a specific impulse control disorder that encompasses behaviors related to blood desires such as seeing blood, bloodletting, and blood‐drinking (Kandeğer et al., 2021).

Given the limited data available in the existing literature and the observations made by experts who work closely with individuals exhibiting nonsuicidal self‐injury (NSSI) have prompted the authors to undertake the following research objectives: (1) What is the prevalence of hemomania behaviors among individuals who engage in NSSI? (2) Are hemomania associated with symptoms such as depression, anxiety, impulsivity, suicidal tendencies, and comorbid psychiatric disorders? (3) Can hemomania be classified as an impulse control disorder accompanied by NSSI behavior and resulting in destructive consequences? To explore this possibility, can the specific diagnostic criteria for hemomania be adapted from the diagnostic criteria of NSSID and pyromania, which is a destructive impulse control disorder, both in DSM 5? In summary, this study aimed to investigate the prevalence of hemomania behaviors and diagnosis and associated clinical factors in individuals with NSSI.

## 2 MATERIALS AND METHODS

### 2.1 Participants and procedure

This cross‐sectional observational study recruited adult psychiatry outpatients aged 18–65 years who engage in NSSI and applied to the Department of Psychiatry, Selçuk University, between June 2022 and January 2023. Following the definition of Current NSSI in DSM‐5, individuals who presented at the psychiatric outpatient clinic and currently exhibited at least one NSSI behavior as part of their clinical presentation were included in the study. During the same evaluation, NSSI behaviors were screened using the Inventory of Statements About Self‐Injury (ISAS). Individuals with behavioral problems associated with intellectual disability, and elevated psychotic symptoms were excluded. Participants with intellectual disability (*n* = 3) and those who did not complete the scales (*n* = 5) were excluded from the study.

Individuals with NSSI who voluntarily agreed to participate in the study were referred for clinical evaluation. The evaluations were conducted by the same experienced psychiatrist (A.K, first author) who possesses 6 years of specialized experience in psychiatry. First, sociodemographic data, a history of suicide attempts, and the use of alcohol, substances, and cigarettes were recorded. Diagnostic evaluations were performed using the Structured Clinical Interview for DSM‐5 Disorders—Clinician Version (SCID‐5), allowing for the determination of both main and comorbid psychiatric diagnoses, and personality disorders as well (Elbir et al., 2019). During the clinical evaluation, patients were also queried regarding the presence of hemomania behaviors (Table 1) and subsequently assessed according to the proposed hemomania criteria which were adapted from the DSM 5 diagnostic criteria of pyromania and NSSID (Table 2). Afterward, participants completed the Barratt Impulsiveness Scale Short Form (BIS‐SF) and the Depression Anxiety Stress Scale (DASS).

**TABLE 1:** Questions for examining *hemomania* behaviors for last year.

**Questions for examining an individual's desire to see/watch their own blood**.
‐During self‐injury, is it important to you to see your blood, to bleed? Would you like to see your blood in particular?
‐How often do you wish to see/watch your blood?
‐What function might it have for you to see/watch your blood?
**Questions for examining an individual's desire to drain, collect, or bloodlet their own blood**.
‐Have you ever intentionally drawn your own blood, either by using a cannula, syringe, or other means of bloodletting?
‐How often do you drain or collect your blood through a syringe, cannula, or anything?
‐What function might it have for you to drain or collect blood from yourself?
**Questions for examining an individual's desire to drink their own blood**.
‐Have you ever tasted/sucked your own blood through forms of self‐injury?
‐Have you ever drunk your own blood that was collected or drained through forms of self‐injury?
‐How often do you drink your blood that was collected or drained through forms of self‐injury?
‐What function might it have for you to drain or collect blood from yourself?

**TABLE 2:** The proposed *Hemomania* criteria that were adapted from the DSM 5 diagnostic criteria of pyromania and NSSID.

In the last year, the individual has, on 5 or more days engaged in *hemomania* behavior(s) as manifested by at least one of the following: Recurrent inability to resist impulses to see/watch one's own blood through self‐injury behaviors, especially with self‐cutting. Recurrent inability to resist impulses to drain/collect one's own blood through self‐injury behaviors, such as bloodletting with a syringe or cannula. Recurrent inability to resist impulses to drink one's own blood through self‐injury behavior, by sucking or drinking the collected blood.
B. Heightened tension or arousal immediately before engaging in *hemomania* behaviors.
C. Pleasure, gratification, or relief during or after engaging in *hemomania* behaviors.
D. *Hemomania* behaviors result in significant distress or impairment in social, occupational, or other important areas of functioning.
E. *Hemomania* behaviors are not associated with suicidal intent and cannot be better explained by symptoms of another mental disorder (for example, this behavior should not be associated with behavioral problems in individuals with intellectual disabilities and should not be considered a response to delusions or hallucinations.).

NSSID, nonsuicidal self‐injury disorder.

The study analysis was conducted with the data of 130 participants who successfully completed the study protocol. This study was granted ethical approval by the Selçuk University Faculty of Medicine Local Ethics Committee (Decision Number: 2021/260).

### 2.2 Measurements

Inventory of Statements About Self‐Injury (ISAS): The ISAS, a two‐part scale developed by Klonsky and Glenn (2009), was validated and made reliable for Turkish use by Bildik et al. (2013). The study employed only the first part of the inventory to screen individuals with NSSI. This section inquiries about the lifetime frequency of 12 intentional nonsuicidal self‐harming behaviors. Participants are asked to indicate how often they engage in each behavior.

Short Form of Barratt Impulsiveness Scale (BIS‐SF‐11): BIS‐SF‐11 was developed by Patton and Stanford in 1995 as a 30‐item 4‐point Likert scale to measure individuals' impulsivity levels (Patton et al., 1995). Tamam et al. (2013) conducted an adaptation study by shortening this 30‐item scale. In the adaptation study, the internal consistency reliability coefficient (Cronbach's Alpha) for the total 15‐item scale was found to be 0.82.

Depression Anxiety Stress Scale‐21 (DASS‐21): DASS‐21 was developed by Lovibond and Lovibond (1995) to assess three components of negative affective states (Depression, Anxiety, Stress). The scale consists of 42 items and is based on a 4‐point Likert scale (0 = Not at all, 3 = Very much) to measure how well each statement describes the individual's feelings. Antony et al. (1998) created a short form of the scale comprising 21 items, known as DASS‐21, which has been found to be a valid and reliable measurement tool. The adaptation of DASS‐21 into Turkish was carried out by Yıldırım et al. (2018). Instead of obtaining a total score from the scale, separate scores are calculated for each of the three subscales (Depression, Anxiety, Stress). Possible scores for each subscale range from 0 to 21. An increase in the score indicates that the individual experiences the corresponding emotional state more intensely.

### 2.3 Analyses

Data entries were input using the SPSS 22 software package. Descriptive statistics were employed to analyze the prevalence of behaviors or diagnoses. After completing normality tests, we used the independent *t*‐test and Mann–Whitney *U* test to compare numerical data between groups, depending on whether the variables were normally distributed. The chi‐square test was utilized to compare categorical data. Subsequently, a logistic regression analysis was conducted using the backward stepwise method to investigate factors associated with the diagnosis of hemomania in bivariate analyses. Age and gender were also controlled in this regression model since younger age and being women were reported as risk factors for NSSI (Muehlenkamp et al., 2012; Ose et al., 2021; Swannell et al., 2014). Hosmer–Lemeshow goodness‐of‐fit statistics were employed to assess model fit. The presence of multicollinearity was assessed by examining the correlation matrix, and no correlation > 0.5 was found among any two independent variables. A 5% type‐1 error level was used to determine statistical significance, with a significance threshold set at *p* < .05.

## 3 RESULTS

The mean age of 130 individuals with NSSI included in the study was 25.28 ± 8.52 (range 18–58), and 63.8% (*N* = 83) were female. The most commonly reported types of self‐injury were banging/hitting self at 66.9%, cutting at 41.5%, hair pulling at 41.5%, and pinching at 32.3%, respectively. 75.4% of the participants engaged in more than one NSSI behavior.

**FIGURE 1:**
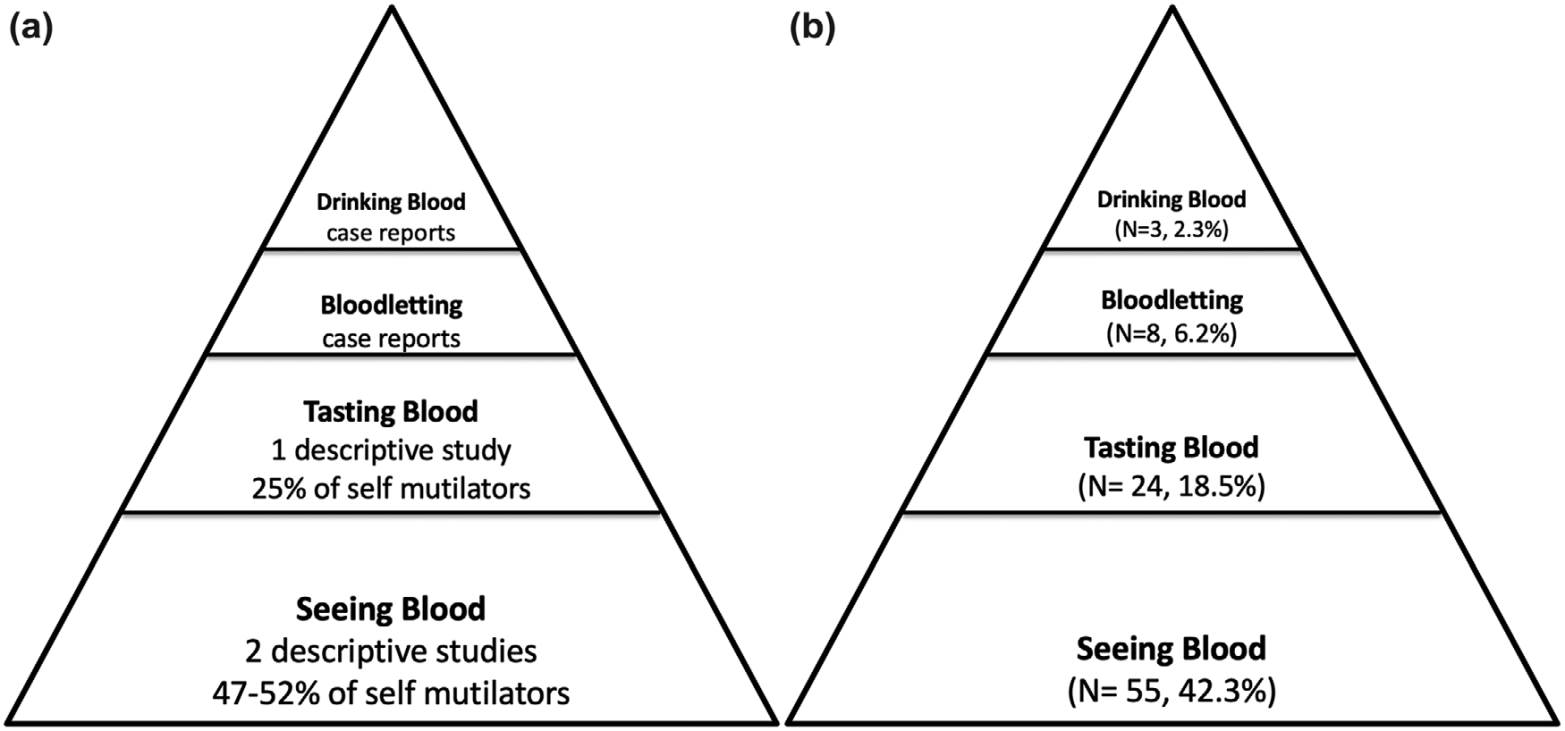
The prevalence of hemomania behaviors in individuals with NSSI (*N* = 130), which we estimated based on data obtained from the literature in our previous report (Kandeğer et al., 2021) (a) and gathered in this study (b).

Out of the participants included in the study who engaged in NSSI, 43.1% (*N* = 56) displayed at least one of the hemomania behaviors within the past year. The prevalence of hemomania behaviors in individuals with NSSI, which we estimated based on data obtained from the literature in our previous report (a) and gathered in this study (b), was illustrated in Figure 1.

On the other hand, 30% (*N* = 39) of the participants met the diagnosis of hemomania, which may be a specific impulse control disorder, according to hemomani diagnostic criteria adapted from the NSSID and pyromania diagnostic criteria in DSM 5. Participants were divided into two groups according to the presence and absence of hemomania. The group with hemomania had more comorbid psychiatric disorders (*t* = −3.27; *p* = .001), more NSSI types engaged (*Z* = −3.63; *p* < .001), a history of suicide attempt (*χ* ^2^ = 6.69; *p* = .010), a higher number of suicide attempts (*Z* = −4.21; *p* < .001) and had higher scores for depression (*t* = −3.05; *p* = .003), anxiety (*t* = −2.65; *p* = .010), stress (*t* = −2.66; *p* = .009), and impulsivity (total score: *Z* = –4.14, *p* < .001; nonplanning: *Z* = –3.06, *p* = .002; motor impulsivity: *Z* = –3.58, *p* < .001; attention impulsivity: *Z* = –3.51, *p* < .001) than those without. The demographic and clinical variables between groups were presented in Table 3.

**TABLE 3:** Comparison of demographic data and means ± standard deviations of measurement tools between groups.

		NSSI with hemomania *N* = 39		NSSI without hemomania *N* = 91		*t*/*Z* or *χ* ^2^	*p*
Age (years)^a^	(Mean, SD)	23.46	6.74	26.07	9.10	1.81	.074
Gender (Woman)^b^	(*N*, %)	28	71.8	55	60.4	1.53	.217
Education (years)^a^	(Mean, SD)	10.03	3.92	10.67	3.50	0.95	.345
Smoking^b^	(*N*, %)	30	76.9	70	76.9	–	1.000
Alcohol consumption^b^	(*N*, %)	24	61.5	50	54.9	1.51	.469
Substance use history^b^	(*N*, %)	13	33.3	36	39.6	2.74	.254
Comorbid psychiatric disorder^a^	(Mean, SD)	3.08	1.12	2.37	1.11	–3.27	**.001**
Suicide attempt in history^b^	(*N*, %)	30	76.9	44	52.4	6.69	**.010**
Number of suicide attempts in history^c^	(Mean, SD)	4.00	4.48	1.29	1.97	–4.21	**<.001**
Number of engaged NSSI types^c^	(Mean, SD)	3.58	1.69	2.37	1.58	–3.63	**<.001**
Depression^a^, ^d^	(Mean, SD)	17.08	4.21	14.53	5.09	–3.05	**.003**
Anxiety^a^, ^d^	(Mean, SD)	16.03	3.59	13.87	5.28	–2.65	**.010**
Stress^a^, ^d^	(Mean, SD)	36.20	8.42	14.73	5.22	–2.66	**.009**
Total impulsivity scores^c^, ^e^	(Mean, SD)	43.39	7.41	35.96	8.19	–4.14	**<.001**
Nonplanning^c^, ^e^	(Mean, SD)	14.64	3.42	12.39	3.91	–3.06	**.002**
Motor impulsivity^c^, ^e^	(Mean, SD)	14.22	3.45	11.73	3.36	–3.58	**<.001**
Attention impulsivity^c^, ^e^	(Mean, SD)	14.53	3.39	12.08	3.48	–3.51	**<.001**

*Note*: Bold values denote statistical significance.

^a^ Independent sample *t*‐test.

^b^ Chi‐squared test test.

^c^ Mann–whitney *U* test.

^d^ Subscores of Depression Anxiety Stress‐21 Scale.

^e^ Total and subscales scores of Short Form of Barratt Impulsiveness Scale

NSSI: nonsuicidal self‐injury.

**FIGURE 2:**
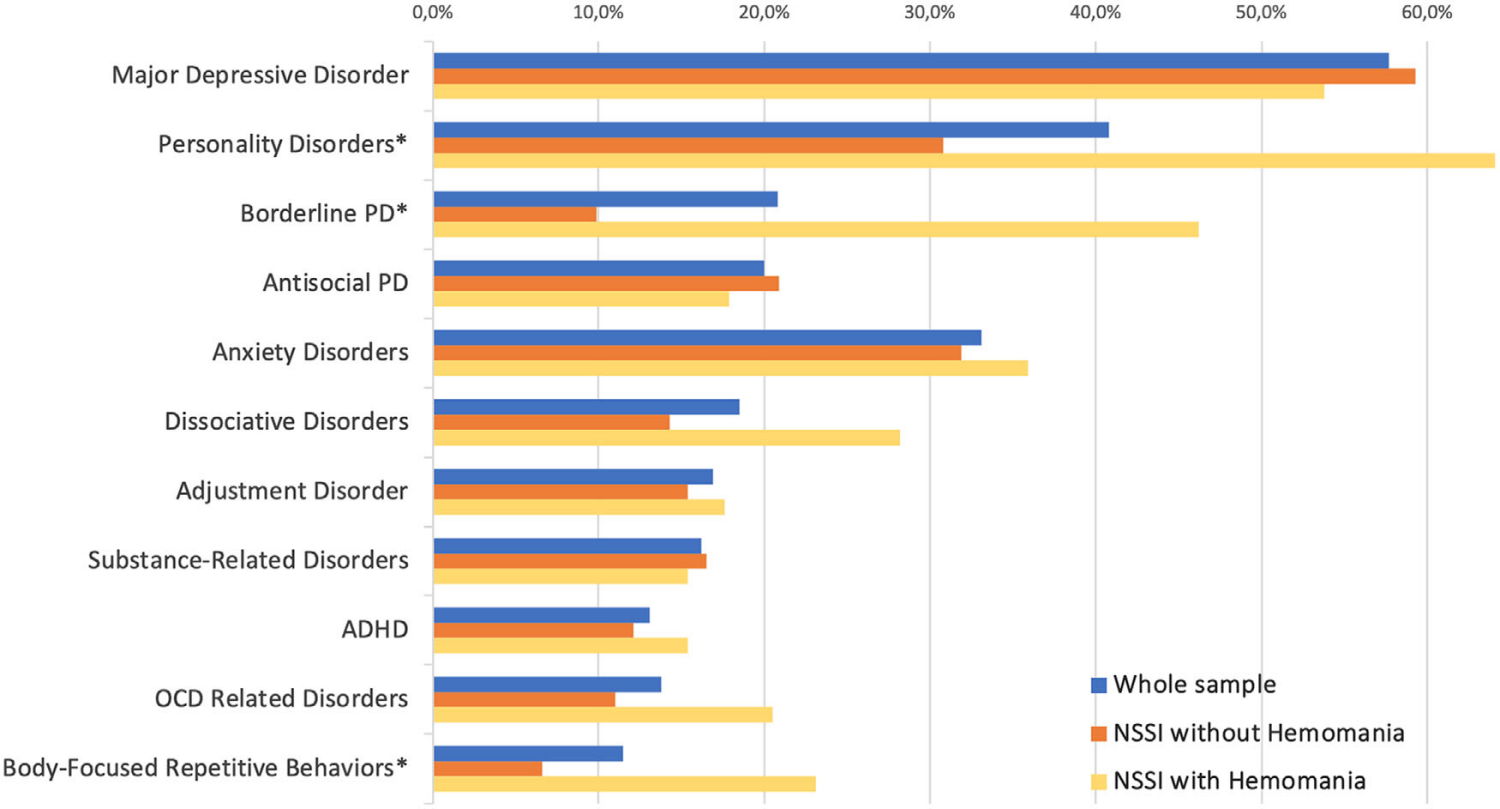
Schematized most prevalent comorbid psychiatric disorders within both the entire sample and groups. *Denotes statistically significance; PD = Personality Disorder; ADHD = Attention Deficit Hyperactivity Disorder.

The prevalent psychiatric disorders in the sample included major depressive disorder (57.7%), personality disorders (40.8%), anxiety disorders (33.1%), and dissociative disorders (18.5%). Figure 2 illustrates a comparison of the prevalent comorbid psychiatric conditions among the groups. Borderline personality disorder (*χ* ^2^ = 21.82; *p* < .001), as well as body‐focused repetitive behaviors (BFRB; hair‐pulling disorder, skin‐picking disorders, nail biting) (*χ* ^2^ = 7.27; *p* = .007), exhibited a higher statistical prevalence within the hemomania group. Additionally, dissociative disorders (*χ* ^2^ = 3.51; *p* = .061) were more prevalent in the hemomania group, although the difference did not reach statistical significance.

**FIGURE 3:**
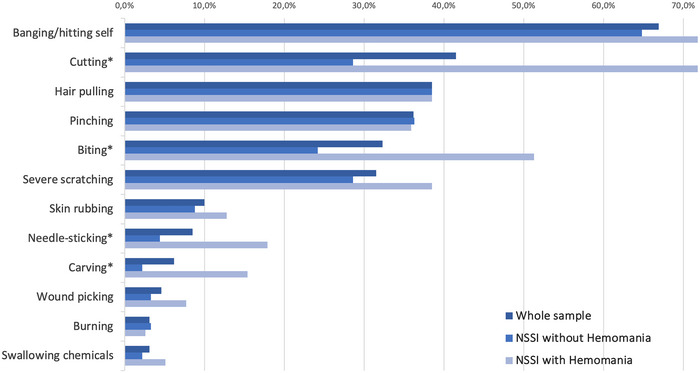
Schematized prevalence of nonsuicidal self‐injury behaviors was questioned with the Inventory of Statements About Self‐Injury within both the entire sample and groups. *Denotes statistically significance.

When we compared the prevalence of NSSI behaviors between groups, cutting (*χ* ^2^ = 21.00; *p* < .001), biting (*χ* ^2^ = 9.17; *p* = .002), needle‐ticking (*χ* ^2^ = 6.47; *p* = .011), and carving (*χ* ^2^ = 8.22; *p* = .004) were significantly more common in the group with hemomania than in the group without. Figure 3 schematically represents the prevalence of NSSI behaviors in both the entire sample and the respective groups.

Finally, age, gender, and factors that varied among two groups in bivariate analysis were included as independent variables for the logistic regression analysis. A backward stepwise method was run, aiming to identify predictors linked to hemomania diagnosis. Thus, numerical variables such as age, number of comorbid psychiatric conditions, number of suicide attempts, number of engaged NSSI behaviors, as well as scores for impulsivity, depression, anxiety, and stress, were included. Categorical variables included gender (male/female), diagnoses of borderline personality disorder, body‐focused repetitive behaviors, and dissociative disorders (which was nearly significant). Additionally, the presence of NSSI behaviors, including cutting, biting, needle‐sticking, and carving, were included. The backward stepwise method was employed in 12 steps, revealing that the most significant factors associated with the diagnosis of hemomania were the number of suicide attempts (Odds  =  1.28; *p*  =  .010; 95% confidence interval  =  1.061−1.534), impulsivity severity (Odds  =  1.07; *p*  =  .041; 95% confidence interval = 1.003−1.138), diagnoses of borderline personality disorder (Odds  =  4.76; *p*  =  .006; 95% confidence interval  =  1.557−14.548), and body‐focused repetitive behavior (Odds  =  10.141; *p*  =  .002; 95% confidence interval  =  2.378−43.245). Hosmer–Lemeshow demonstrated valid goodness‐of‐fit statistics in all steps. The model accounted for 32.6% (Cox and Snell *R* ^2^) to 46.8% (Nagelkerke *R* ^2^) of the variance in hemomania diagnosis, achieving an overall correct classification of the cases with a rate of 81.5%. The results of the analysis were presented in Table 4.

**TABLE 4:** Logistic regression analyses for predictors of hemomania diagnosis.

	ODDS	95%CI	*p*
Number of suicide attempts	1.28	1.061–1.534	**.010**
Impulsivity severity^a^	1.07	1.003–1.138	**.041**
Self‐cutting behavior	0.35	0.121–1.029	.056
Borderline personality disorder	4.76	1.557–14.548	**.006**
Body‐focused repetitive behaviors	10.141	2.378–43.245	**.002**

*Note*: This presents the final step of a 12‐step backward stepwise model utilized in logistic regression analysis to identify predictors of hemomania diagnosis. The model explained between 32.6% (Cox and Snell *R* ^2^) and 46.8% (Nagelkerke *R* ^2^) of the variance in hemomania diagnosis, correctly classifying 81.5% of cases overall. Included variables in the model: age, number of comorbid psychiatric conditions, number of suicide attempts, number of engaged NSSI behaviors; scores for impulsivity, depression, anxiety, and stress; gender (male/female); having diagnoses of borderline personality disorder, body‐focused repetitive behaviors, and dissociative disorders; the presence of certain NSSI behaviors such as including cutting, biting, needle‐sticking, and carving. Bold values denote statistical significance.

^a^ Total scores of Short Form of Barratt Impulsiveness Scale.

## 4 DISCUSSION

This study investigates the possibility that the diagnosis of hemomania in individuals with NSSI may indicate a specific impulse control disorder. Among individuals with NSSI, the prevalence of hemomania behaviors was 43.1%, while the prevalence of a hemomania diagnosis stood at 30%. The group diagnosed with hemomania exhibited a higher number of comorbid psychiatric disorders, a greater prevalence of suicide attempts, and more severe symptoms of depression, anxiety, and stress compared to the group without a hemomania diagnosis. Furthermore, significant distinctions emerged between the two groups in terms of both the NSSI behaviors displayed by individuals and the prevalence of comorbid psychiatric disorders.

When the demographic characteristics of individuals with NSSI are examined, it is seen that the prevalence is especially high in adolescents and young adults. The mean age of the participants included in our study was approximately 25.28 (range 18–58), and the median was 22. A recent systematic review underscores that NSSI is more prevalent during adolescence, with the typical onset occurring around the ages of 12–14 (Cipriano et al., 2017). Regarding gender distribution, our study observed a prevalence of 63.8% among women. This proportion aligns harmoniously with the consistent data reported in the existing literature (Ose et al., 2021). It is obvious that adolescent and young adult women are also the target group for early intervention in the behaviors of NSSI.

The most common NSSI behaviors used by the participants were banging/hitting, cutting, hair pulling, pinching, biting, and severe scratching, in line with the literature. At least 75.4% of participants were engaged with more than one NSSI behavior, nearly matching the rate reported in the literature of 78% (Favazza & Conterio, 1988; Klonsky et al., 2014).

### 4.1 Hemomani behaviors and diagnosis

In the main aim of our study, hemomania behaviors were determined as seeing blood, tasting blood, bloodletting, drinking blood, and their prevalences were determined as 42.3%, 18.5%, 6.2%, and 2.3%, respectively. These rates confirmed the pyramidal prevalence distribution we estimated in our previous report (Kandeğer et al., 2021). In line with this, while exceptions may exist, the desire to see blood emerges as the foundational element underpinning hemomania behaviors. Our proposed perspective maintains that the act of observing blood serves as a precursor to the behaviors higher up the prevalence pyramid. As one ascends the pyramid, the intensity of destructiveness amplifies, concomitant with a decline in prevalence (Figure 1).

A combination of empirical studies, case series, and observations of the authors of this study collectively point toward the prevalence of hemomania behaviors. These behaviors are frequently employed for the purposes of alleviating stress and tension, and experiencing pleasure. Additionally, it has been observed that these behaviors are linked to disorders characterized by heightened impulsivity, such as borderline personality disorder and bulimia nervosa. This cumulative evidence prompted our endeavor to formulate a distinct conceptual framework for an impulse control disorder closely associated with hemomania (Robbins et al., 2012). Considering the accompanying NSSI behaviors and the destructive nature of this phenomenon, when the participants were evaluated according to the diagnostic criteria adapted from the NSSID and pyromania diagnostic criteria, 30% of the participants met the diagnosis of hemomania. In subsequent sections of the article, the primary symptoms potentially linked to hemomania will be discussed, starting with impulsivity.

### 4.2 Hemomania and impulsivity

In our study, we observed that the group with hemomania exhibited higher impulsivity scores compared to the group without hemomania. Furthermore, our regression analysis revealed that impulsivity plays a significant role as a risk factor for the diagnosis of hemomania, even when considering all associated factors with hemomania.

While it is traditionally known that nonsuicidal self‐injury (NSSI) is associated with impulsivity, there are some complex findings in this regard. For instance, in a review and meta‐analysis, authors have suggested that impulsivity is better measured using neurocognitive tests as opposed to self‐report instruments (Hamza et al., 2015). In one study, no significant difference in BIS‐11 scale scores was found between adolescents engaging in NSSI and healthy controls, although it should be noted that this study had a small sample size (Liu et al., 2017). It is important to acknowledge that impulsivity is not a singular construct. Meta‐analysis results have indicated that self‐reported impulsivity is particularly associated with the negative urgency domain, which may explain self‐injury behaviors as a means of reducing distress and seeking relief (Hamza et al., 2015). Additionally, it may point to difficulties in emotion regulation, which will be discussed in the following section.

While impulsivity is often linked to NSSI, it may not necessarily represent its core or cardinal symptom. When comparing NSSI individuals without hemomania to those with hemomania, it becomes evident that individuals with hemomania tend to have higher impulsivity severity. Moreover, our regression analysis showed that impulsivity is associated with all related (suicide attempts, borderline personality disorder, BFRBs) factors in hemomania (Grant et al., 2022; Gunderson et al., 2018; Maraz et al., 2017; Mirabella et al., 2023). These findings suggest that impulsivity may be a more significant issue in individuals with hemomania than in those without hemomania.

There was a higher prevalence of borderline personality disorder within the hemomania group. This association can be attributed to impulsivity being a fundamental symptom of borderline personality disorder, and this disorder, along with other impulse control disorders, tends to exhibit a greater occurrence (Gunderson et al., 2018). Nevertheless, dissociative disorders, which are characterized by impulsivity, also displayed a higher occurrence within the hemomania group, and very slightly missing the significance level (Zlotnick et al., 1997). The constrained sample size might have contributed to preventing this relationship from reaching the threshold of statistical significance.

BFRBs (hair‐pulling, skin picking, nail biting) are diagnoses characterized by a high impulsivity pattern, leading to ongoing debates about their position on the impulsivity‐compulsivity axis (Maraz et al., 2017; Robbins et al., 2012). Functional connectivity studies on BFRBs presented findings on the dysfunction of reward processing and dysregulated reward circuitry (Grant et al., 2022). A recent study involving 194 participants has reported that skin picking behavior primarily serves as an impulsive behavior that provides temporary relief from intense emotions (Schienle et al., 2018). This finding coincides with the role of hemomania and underlines the significance of emotion regulation.

### 4.3 Hemomania and emotion dysregulation

While impulsivity takes precedence initially, the subsequent criterion for diagnosing hemomania involves the manifestation of emotional dysregulation. Emotional dysregulation is a characteristic frequently observed in borderline personality disorder, which holds a higher prevalence within the hemomania group. Additionally, it also tends to be prevalent in dissociative disorders, a category that tends to be more frequent among individuals with hemomania (Brand & Lanius, 2014). The utilization of BFRBs as a mechanism for regulating emotions, particularly among adolescents, intersects with the underlying intent of hemomania (Ricketts et al., 2022; Snorrason et al., 2010). As stated in the diagnostic criteria, individuals with hemomania use these behaviors to reduce tension and relief. This may be a maladaptive behavior such as BFRBs used to regulate compulsive emotions heightened by borderline features and dissociative symptoms. A recent study of 53 young people also showed that those with BFRBs had higher impulsivity, lower distress tolerance, and more negative emotions (Ricketts et al., 2022).

### 4.4 Hemomania and internalizing symptoms

In our study, the group with hemomania exhibited shared characteristics with borderline features, including heightened negative emotions and an increased incidence of suicide attempts. Hemomania appears to demonstrate a stronger propensity for association with traits such as impulsivity, emotional dysregulation, and internalizing symptoms. Negative emotions such as anxiety and depression, low self‐esteem, and increased suicidal behavior, which manifest as symptoms directed inwardly within individuals are defined as internalizing symptoms (Achenbach et al., 2016). Despite the pronounced presence of impulsivity, it may also explain that the high levels of externalizing symptoms often associated with conditions like ADHD, ASPD, and substance‐related disorders (including traits like aggression and hyperactivity) do not appear to be markedly elevated within the hemomania group.

On the other hand, people who fail to introject their conflicts and tend to internalize their symptoms may direct their impulses and anger toward their own bodies (suicidal behaviors) and may try to regulate their emotions through their own bodies (hair pulling, skin picking, nail biting, and hemomania behaviors). A recent study in which structural equation model analysis was conducted also suggested that emotion dysregulation associated with impulsivity increases NSSI and suicide frequency through internalizing symptoms (Kranzler et al., 2016).

To embody this explanation, individuals with BFRBs do not limit their interaction with the targeted body part (hair, nail, skin) to mere plucking; but more rarely watch, chew, and eat it (Snorrason et al., 2011; Snorrason et al., 2012). From a similar perspective, hemomania behaviors share parallels with this process. The desire of individuals to see their own blood can also progress to the desire to drink it. Although it is unclear how these behaviors relieve tension and provide pleasure, our previous report discussed the psychodynamic explanation for hair‐pulling disorder. According to this, individuals who have these disorders had a failure in introjection in primary relationships. Introjection of an object (e.g., hair, nail, skin, or blood) eliminated from the self may be an attempt to compensate for or make sense of the failure of introjection in childhood experiences and relations (Kandeğer et al., 2021; Saya et al., 2018).

### 4.5 Hemomania and NSSI behaviors

When comparing NSSI behaviors between groups, the hemomania group exhibited higher rates of cutting, biting, needle‐ticking, and carving. These behaviors all pose a risk to skin integrity, potentially facilitating blood‐related actions. This suggests that individuals with hemomania might be primarily driven by a desire to access blood. Interestingly, cases with hemomania in our previous report who acquired the skill to extract blood using a cannula showed a tendency to stop self‐cutting. This suggests that the pursuit of blood might explain the preference for NSSI methods that involve a higher likelihood of encountering blood (Kandeğer et al., 2021). However, further quantitative analysis is needed to solidify this hypothesis.

### 4.6 Limitations

The present study, while providing valuable insights, has certain limitations. Notably, while psychiatric diagnoses were assessed using the SCID‐5, several other metrics relied on self‐report instruments. Furthermore, our study exclusively involved adults within the specified age group, excluding adolescents and individuals aged 12–18. It is worth noting that both NSSI and hemomania often emerge at younger ages, which may limit the generalizability of our findings to young adults. Additionally, our investigation into NSSI and hemomania behaviors adopted a cross‐sectional design, and we did not analyze the duration of these behaviors, potentially affecting our findings. Another important consideration concerns the assessment of hemomania behaviors and the corresponding diagnostic criteria, which were evaluated using adapted forms developed by the researchers. To establish the robustness and applicability of these criteria, it is crucial to undertake validation efforts with a larger and more diverse cohort. Finally, despite its utility, backward stepwise regression model's reliance on statistical significance poses a limitation, particularly pronounced in the context of causal effects in big data scenarios. It may overlook genuinely influential variables while erroneously incorporating insignificant ones, leading to a model that performs well within the sample but fails to generalize effectively out‐of‐sample (Smith, 2018). However, since our study was a cross‐sectional pilot study and focused on identifying the most relevant factors rather than establishing causality, this limitation may have been mitigated.

## 5 CONCLUSIONS

The prevalence and adverse effects of NSSI among adolescents and young adults are a cause for concern. This study suggests that hemomania may be a specific impulse control disorder with destructive consequences. Among participants exhibiting NSSI, the adapted hemomania diagnostic criteria proved effective in distinguishing individuals with hemomania from those without, particularly in terms of comorbid psychiatric conditions, suicide attempts, depression, anxiety, stress levels, impulsivity severity, and the specific method of NSSI employed. The relationship between hemomania and traits like impulsivity, emotion dysregulation, and internalizing symptoms (such as negative emotions and suicidal behavior) has been thoroughly examined. However, it is important to note that further clinical investigations involving larger participant samples are necessary to validate the assessment tools used and to gain a more comprehensive understanding of the distinctive aspects associated with hemomania.

## AUTHOR CONTRIBUTIONS

**Ali Kandeger**: Conceptualization; methodology; investigation; formal analysis; supervision; writing―original draft; writing―review and editing; visualization; software; resources. **Omer Faruk Uygur**: Methodology; data curation; investigation; validation; formal analysis; writing―original draft; software; project administration. **Emine Yavuz Ataslar**: Data curation; validation; formal analysis; visualization; software. **Furkan Cınar**: Visualization; data curation; validation. **Yavuz Selvi**: Supervision; writing―review and editing; validation; project administration.

## FUNDING

The authors have no funding to declare.

## CONFLICT OF INTEREST STATEMENT

The authors have no conflicts of interest to declare.

### PEER REVIEW

The peer review history for this article is available at https://publons.com/publon/10.1002/brb3.3475.

## Data Availability

The data that support the findings of this study are not publicly available due to ethics restrictions but available from the corresponding author upon reasonable request with individual permission from local institutions ethics board.
